# Associations with early vomiting when using intranasal fentanyl and nitrous oxide for procedural sedation in children: A secondary analysis of a randomised controlled trial

**DOI:** 10.1111/1742-6723.14497

**Published:** 2024-09-13

**Authors:** Emmanuelle Fauteux‐Lamarre, Stephen Hearps, Michelle McCarthy, Nuala Quinn, Andrew Davidson, Donna Legge, Katherine J Lee, Greta M Palmer, Sandy M Hopper, Franz E Babl

**Affiliations:** ^1^ Emergency Department Cork University Hospital Cork Ireland; ^2^ Murdoch Children's Research Institute Melbourne Victoria Australia; ^3^ Emergency Department The Royal Children's Hospital Melbourne Victoria Australia; ^4^ Emergency Department Children's Health Ireland at Temple Street Dublin Ireland; ^5^ Department of Paediatrics, Faculty of Medicine, Dentistry and Health Sciences University of Melbourne Melbourne Victoria Australia; ^6^ Department of Anaesthesia and Pain Management The Royal Children's Hospital Melbourne Victoria Australia; ^7^ Melbourne Children's Trials Centre Melbourne Victoria Australia; ^8^ Department of Pharmacy The Royal Children's Hospital Melbourne Victoria Australia

**Keywords:** fentanyl, nitrous oxide, ondansetron, procedural sedation, vomiting

## Abstract

**Objective:**

Intranasal (IN) fentanyl and nitrous oxide (N_2_O) can be combined to provide procedural sedation and analgesia to children. This combination is advantageous because of rapid onset of action and non‐parenteral administration, but is associated with increased vomiting. We sought to describe the associations of demographic and procedural factors with early vomiting when using this combination in children.

**Methods:**

This was a planned secondary analysis of a randomised controlled trial comparing the effect of oral ondansetron *versus* placebo at a single paediatric hospital. Children aged 3 to <18 years with planned procedural sedation with IN fentanyl and N_2_O were randomised to receive oral ondansetron or placebo prior to N_2_O administration. Vomiting was defined as early if occurring during or up to 1 h after N_2_O delivery. We assessed the relationship between early vomiting, demographic and procedural characteristics.

**Results:**

Participants were recruited between October 2016 and January 2019 and 62 out of 436 (14%) had early vomiting. The risk of early vomiting was 30% higher with higher total dose of fentanyl, risk ratio = 1.3 (95% confidence interval = 1.004–1.59). There was little evidence of a relationship between the occurrence of early vomiting and sex, age, weight, type of procedure, fasting duration, time between fentanyl administration and start of procedure, and procedure duration.

**Conclusion:**

We found that higher doses of IN fentanyl were associated with higher risk of early vomiting when administered with N_2_O in children. Other factors did not appear to be associated with vomiting.

**Key findings**

1. We found higher risk of early (within 0–1 h) vomiting with higher total doses of intranasal fentanyl (non‐procedural and procedural combined).
2. Clinicians may consider the use of non‐opioid adjunctive analgesics to avoid higher total doses of intranasal fentanyl.
3. Areas for future research might include comparing nitrous concentrations (50% *vs*. 70%) with respect to vomiting occurrence when combined with IN fentanyl.

## Introduction

Procedural sedation with nitrous oxide (N_2_O) can be augmented with intranasal (IN) fentanyl to provide enhanced pain relief to children undergoing noxious interventions where the use of N_2_O alone may be considered insufficient.^1^, ^2^, ^3^ This combination of agents has a few advantages: the onset of action is rapid, intravenous access is not required and the recovery period is brief.^2^, ^3^ However, this sedation regimen is associated with a higher vomiting occurrence than N_2_O alone, 13–30% *versus* less than 6%.^2^, ^3^, ^4^, ^5^, ^6^, ^7^, ^8^ Intraprocedural vomiting is undesirable for numerous reasons: it is disruptive to the process, potentially distressing to patient and family and may increase the risk of pulmonary aspiration.^9^, ^10^ Ultimately, it may necessitate abandonment of the procedure. However, we do not know which children are at risk of vomiting with this sedation strategy. Identifying children at greatest risk of vomiting may drive decisions around the use of prophylactic antiemetic agents or whether or not to use this sedation strategy. In our setting, this combination is mainly employed for forearm fracture reductions, as was the case in other institutions that have reported on this sedation regimen.^2^, ^3^, ^4^, ^11^, ^12^

We conducted a randomised controlled trial (RCT) of oral ondansetron *versus* placebo to determine the efficacy of ondansetron to decrease early (within 0–1 h) and any vomiting within 24 h of procedural sedation with the combination of IN fentanyl and N_2_O. In this pre‐planned secondary analysis, we aimed to determine which demographic, clinical and procedural factors were associated with early vomiting. Knowledge of particular patient groups or factors identified as increasing the risk of early vomiting would help clinicians optimise sedation regimens, counsel patients and their families when obtaining informed consent for the procedure, and prepare for the occurrence of vomiting during the procedure.

## Methods

This was a pre‐planned secondary analysis of an RCT of oral ondansetron *versus* placebo. A short summary of the study protocol is presented here. The complete protocol^13^ and primary analysis results^5^ of the RCT are presented elsewhere.

### Study design, setting and patients

This was a double‐blind, placebo‐controlled superiority trial of pre‐procedural oral ondansetron to prevent vomiting in children receiving procedural sedation with the combination of IN fentanyl and N_2_O. A sublingual placebo was unavailable when the trial was developed. The study was conducted at a single tertiary care paediatric ED, with an annual census of 85 000.

Children aged 3 to <18 years of age with planned procedural sedation with IN fentanyl and N_2_O were eligible for recruitment. We excluded patients with contraindications to receiving IN fentanyl, N_2_O or ondansetron, concomitant head injury, active intercurrent illness causing nausea or vomiting, or planned use of additional sedatives. Younger children were excluded as having additional study drug and placebos doses was not feasible. In this secondary analysis, we have combined patients in the ondansetron and placebo groups.

This RCT received ethics approval from the Royal Children's Hospital Human Research Ethics Committee (reference number 36174A) and was registered with the Australian and New Zealand Clinical Trials Registry (ACTRN12616001213437).

### Patient and public involvement

Patients or the public were not involved in the design, conduct, reporting or dissemination of our research.

### Study procedures

All emergency clinicians received training to identify, recruit and obtain informed consent from participants and their families. Suitable participants were approached by a paediatric emergency doctor or nurse practitioner after clinical assessment, when the need for procedural sedation with IN fentanyl and N_2_O was established. After written consent was obtained, participants were randomised to receive oral ondansetron or placebo (strawberry‐flavoured base suspension) 30–60 min prior to N_2_O administration. Participants with weights of 15–30 kg and >30 kg received 4 mg (5 mL) and 8 mg (10 mL) doses of the study drug, respectively. Children received analgesia according to an analgesic ladder. This included providing paracetamol and ibuprofen where appropriate. Intranasal fentanyl was administered shortly before or after the study drug. The procedural dose of IN fentanyl was 1.5 mcg/kg (max. 75 mcg). The procedural fentanyl dose could be reduced by up to half, according to clinician assessment, where the participant had already received IN fentanyl for pain relief within 30–60 min prior to recruitment. In these cases, clinicians considered several factors when choosing the procedural fentanyl dose: current patient pain score, exact timing since the last fentanyl dose, projected delay before the procedure and personal experience. In our setting, IN fentanyl is normally the only opioid provided in the pre‐hospital and pre‐procedural period for this patient cohort. N_2_O via continuous flow was initiated at 50–70% concentration 30 min post IN fentanyl and titrated to 70% for 3–5 min prior to starting the painful procedure. This high concentration of N_2_O is commonly used at our institution in order to achieve greater sedation depth and analgesia to perform more painful procedures such as fracture reductions.^2^

### Data collection and outcomes

As well as demographic factors (sex, age and weight), we collected data regarding the type of procedure, fasting duration, total fentanyl dose, time between fentanyl administration and start of procedure, and procedure duration as potentially associated with increased early vomiting.

The primary outcome from the trial was early vomiting (yes/no) related to procedural sedation, defined as vomiting occurring from the commencement of N_2_O until discharge from the paediatric ED or within 1 h of the procedure (whichever came first). Vomiting was defined as forceful expulsion of gastric contents from the mouth. Secondary outcomes included: number of vomits and retching during procedural sedation, vomiting post‐discharge (from discharge from the paediatric ED or 1 h after the procedure (whichever came first)) up to 24 h after the commencement of N_2_O, ‘any vomiting’ (defined as early and post‐discharge vomiting combined), procedure duration, procedure abandonment and adverse events, sedation quality as per the treating clinician's subjective assessment and parental satisfaction.

Treating clinicians recorded all necessary procedure and outcome data on the participant's case report form. A study investigator called all families after discharge to inquire about post‐procedural vomiting.

### Statistical analysis

Descriptive statistics are presented for patient demographics, procedural characteristics and outcomes. Data are combined across the two treatment groups as the treatment group was not of interest in this analysis. Categorical characteristics are presented as frequencies and percentages. Child's age is summarised via mean and standard deviation, and average fasting hours are presented as median and interquartile range. Summary statistics of clinical demographic and procedural variables are presented separately by those with early and no early vomiting. Mean group differences between those with early and no early vomiting are calculated for continuous measures, and differences in group proportions for binary variables, with 95% confidence intervals (95% CIs). Unadjusted logistic regression is used to describe the relationship between demographic and procedural characteristics and early vomiting, with results presented as risk ratios (RRs) and their 95% CI applied to those with outcome data available.

## Results

We recruited 442 participants between October 2016 and January 2019. Of the 442 participants, 436 patients had primary outcome data available and were included in this secondary analysis.

The baseline demographic characteristics of the 436 participants included in the analysis are presented in Table 1. The majority of participants underwent procedural sedation with IN fentanyl and N_2_O for a fracture reduction (75%). Most patients were fasted for liquids (91%) and solids (97%) for a minimum of 2 h.

**TABLE 1:** Associations of early vomiting with demographic and procedural characteristics in combined data set (ondansetron and placebo)

	All families	Early vomiting	No early vomiting		
	*n* = 436	*n* = 62	*n* = 374	Diff (95% CI)	RR (95% CI)
Age (years), mean (SD)	8.79 (3.38)	9.52 (3.10)	8.67 (3.41)	0.8 (−0.1 to 1.8)	1.1 (0.995–1.1)
Sex, *n* (%)					
Male	271	42 (16)	229 (85)	3.4 (−3.2 to 10.0)	1.3 (0.8–2.1)
Female	165	20 (12)	145 (88)		
Weight group, *n* (%)					
≥30 kg	216	32 (15)	184 (85)	1.2 (−5.4 to 7.7)	1.1 (0.7–1.7)
15–30 kg	220	30 (14)	190 (86)		
Procedure type, *n* (%)					
Fracture reduction	329	48 (15)	281 (85)	1.5 (−5.9 to 8.9)	1.1 (0.6–1.9)
(None)	107	14 (13)	93 (87)		
Dislocation reduction	17	1 (6)	16 (94)	−8.7 (−20.4 to 3.0)	0.4 (0.1–2.7)
(None)	419	61 (15)	358 (85)		
Splint/cast application	29	4 (14)	25 (86)	−0.5 (−13.5 to 12.5)	1.0 (0.4–2.5)
(None)	407	58 (14)	349 (86)		
Laceration repair	27	5 (19)	22 (81)	4.6 (−10.4 to 19.6)	1.3 (0.6–3.0)
(None)	409	57 (14)	352 (86)		
Abscess drainage	11	2 (18)	9 (82)	4.1 (−19.0 to 27.1)	1.3 (0.4–4.6)
(None)	425	60 (14)	365 (86)		
Other	28	2 (7)	26 (93)	−7.6 (−17.7 to 2.6)	0.5 (0.1–1.9)
(None)	408	60 (15)	348 (85)		
Fasting, *n* (%)^†^					
Fasted ≥1 h liquid	400	58 (15)	342 (86)	−5.5 (−40.7 to 29.7)	0.7 (0.1–4.3)
Fasted ≥2 h liquid	369	58 (16)	311 (84)	12.9 (6.4–19.5)	5.7 (0.8–39.7)
Fasted ≥4 h liquid	219	37 (17)	182 (83)	5.1 (−1.7 to 11.9)	1.4 (0.9–2.3)
Fasted ≥6 h liquid	85	17 (20)	68 (80)	6.9 (−2.4 to 16.1)	1.5 (0.9–2.5)
Fasted ≥2 h solid^‡^	391	59 (15)	332 (85)	15.1 (11.5–18.6)	‐
Fasted ≥4 h solid	282	45 (16)	237 (84)	4.6 (−2.5 to 11.6)	1.4 (0.8–2.5)
Fasted ≥6 h solid	134	22 (16)	112 (84)	2.8 (−4.7 to 10.3)	1.2 (0.7–2.0)
Study related procedures					
Prior non‐procedural fentanyl, *n* (%)	136	22 (16)	114 (84)	2.8 (−4.4 to 10.1)	1.2 (0.8–2.0)
(None)	300	40 (13)	260 (87)		
Procedural fentanyl dose (mcg/kg), mean (SD)	1.43 (0.30)	1.41 (0.24)	1.44 (0.31)	0.0 (−0.1 to 0.1)	0.7 (0.3–1.7)
Total fentanyl dose (mcg/kg), mean (SD)	2.00 (0.99)	2.24 (1.56)	1.96 (0.86)	0.3 (0.01–0.5)	1.3 (1.04–1.6)
Time between last non‐procedural INF and procedure start (min), mean (SD)	156.11 (64.21)	169.77 (68.76)	153.43 (63.26)	16.3 (−13.0 to 45.7)	1.0 (0.998–1.01)
Time between procedural INF and procedure start (min), mean (SD)	42.42 (16.19)	44.55 (16.43)	42.07 (16.14)	2.5 (−1.9 to 6.8)	1.0 (0.995–1.02)
Time between study drug and procedure start (min), mean (SD)	43.76 (17.12)	46.06 (20.47)	43.37 (16.49)	2.7 (−1.9 to 7.3)	1.0 (0.995–1.02)
Duration of sedation procedure (min), mean (SD)	13.77 (6.99)	14.07 (7.36)	13.72 (6.94)	0.4 (−1.6 to 2.3)	1.0 (0.97–1.04)

^†^ Thirty‐one missing.

^‡^ RR not calculable, zero cell count.

Fasting groupings not mutually exclusive.

Diff = percentage difference for categorical measures, mean difference for continuous.

95% CI, 95% confidence interval; INF, intranasal fentanyl; RR, risk ratio; SD, standard deviation.

Of the 436 participants, 62 (14%) had early vomiting related to the procedure (Table 1). Phone follow‐up providing data on vomiting up to 24 h post‐procedure, was available for 400 participants. Of those, 104 (26%) had any vomiting up to 24 h after procedure start.

We found little evidence of an association between sex, age, weight, type of procedure, fasting duration, time between fentanyl administration and start of procedure, and procedure duration and the occurrence of early vomiting (Table 1). A higher total dose of fentanyl (non‐procedural and procedural) was associated with an increased risk of early vomiting, RR = 1.3 (95% CI = 1.004–1.59). This risk ratio signifies a 30% increased risk of vomiting for each 1 mcg/kg increment in total fentanyl dose above the standard 1.5 mcg/kg dosing. Additionally, we found little evidence of an association between the occurrence of any vomiting up to 24 h from procedure start and the above factors.

## Discussion

In this pre‐planned secondary analysis of an RCT of oral ondansetron *versus* placebo to decrease vomiting in children receiving the combination of IN fentanyl and N_2_O for procedural sedation, we found that a higher total dose of fentanyl (non‐procedural and procedural) was associated with an increased risk of early vomiting. There was little evidence of association for any other demographic or procedural characteristics. Interestingly, fasting status was not found to be associated with vomiting.

The occurrence of vomiting found in the current trial (early vomiting 14%; any vomiting up to 24 h 26%) is broadly comparable to two previously reported prospective but smaller observational studies of patients given up to a 70% N_2_O concentration.^2^, ^3^ These two previous studies involved our institution solely or in part and early vomiting occurred in 19.5%^2^ and any vomiting was seen in 29.6%^3^ of patients who had received 70% N_2_O. Two other, larger, studies in a single Swiss centre reported much lower occurrences of any vomiting at 8.7% and 15.4%, respectively (where IN fentanyl recipients numbered *n* = 201^4^ and *n* = 206^12^). Notably, the duration of 70% N_2_O administration in these Swiss studies was much shorter (mean duration of 3.8^4^ and 4.6^12^ min, respectively) than in the current trial (mean duration of 13.7 min).

Of the two previous observational studies involving our institution, one suggested an association between vomiting and a shorter time delay between IN fentanyl and N_2_O delivery.^2^ In that study, 75% of patients who vomited had received their fentanyl dose within 10 min of N_2_O administration. It was this observational data that led us to impose a time delay of 30 min between IN fentanyl and N_2_O in the current trial and no association with vomiting persisted at or beyond this prescribed time delay. The other observational study was performed in a setting where variable concentrations of N_2_O were used as part of their usual clinical practice. It reported lower rates of vomiting when a maximum N_2_O concentration of 50% *versus* 70% (10.5% *vs*. 29.6%).^3^ As the concentration of N_2_O delivered was at the discretion of the providing clinician, it is possible that the two patient groups differed in their demographic characteristics and nature of the procedure (i.e. procedures considered more painful were treated with higher concentrations of N_2_O). Another retrospective observational study, conducted in Spain, of patients receiving IN fentanyl and 50% N_2_O for orthopaedic procedures reported no instances of vomiting in 52 patients.^11^ Despite these smaller studies documenting reduced vomiting occurrence with the lower 50% N_2_O concentration, our ED preferentially uses 70% as the benefit from deeper sedation is believed to outweigh the increased vomiting risk.^8^

In the current trial, we found an increased risk of early vomiting with a higher total dose of fentanyl (non‐procedural and procedural). Higher total doses were administered where patients had received a dose of IN fentanyl pre‐hospital or on arrival to hospital, followed by a further dose 30 min prior to their procedure. Our trial protocol suggested a procedural dose of IN fentanyl of 0.75–1.5 mcg/kg in cases where the patient had received IN fentanyl 30–60 min prior the study drug administration. This adjustment may not be the norm in standard practice where EDs have protocolised dosing, typically of 1.5 mcg/kg of procedural IN fentanyl. Additionally, nausea and vomiting are inherent side effects of opioid administration and may be difficult to mitigate when analgesic efficacy for severe pain is sought.

With the use of IN fentanyl and N_2_O in combination for procedural sedation, the only factor where we found evidence of an association with vomiting was a higher total fentanyl dose. Given this, we recommend that all patients and families should be counselled that early vomiting is common, affecting one in seven children and can occur for an additional one in eight children after discharge from the ED. Another consideration may be to employ non‐opioid agents, such as non‐steroidal anti‐inflammatory drugs via a number of routes, which we did not investigate in the present study.

### Limitations

The current study needs to be framed within its limitations. First, our findings might not easily be generalised to centres where lower concentrations of N_2_O are routinely used or where the procedural profile is sufficiently different. Second, our investigation may not have identified patients at higher risk of vomiting, such as those with known motion sickness or those with higher pre‐procedural pain scores. Third, we may have failed to ascertain contributors to vomiting because of our prescriptive protocol for the timing and dosing of drugs. We also did not collect data on other opioids administered prior to enrolment. In addition, we did not collect data on the missed eligible patients. Finally, the study findings are descriptive in nature; we do not attempt to draw any causal conclusions.

## Conclusions

We found that higher total doses of IN fentanyl were associated with higher risk of early vomiting when administered with N_2_O in children. Other factors did not appear to be associated with vomiting. An area for future investigation may include comparing nitrous concentrations (50% *vs*. 70%, for instance) with respect to vomiting occurrence when combined with IN fentanyl for procedural sedation.

## Data Availability

The authors elect not to share data.
